# Preoperative bladder artery embolization before transurethral resection of large bladder tumors: a propensity score–matched retrospective cohort study

**DOI:** 10.1186/s42155-026-00779-4

**Published:** 2026-10-19

**Authors:** Chengtao Gu, Jun Li, Hao Zheng, Zilong Zhang, Hao Xu, Zhe Zhang, Gang Shen, Long Cheng

**Affiliations:** 1 Department of Interventional and Vascular Surgery, The Fourth Affiliated Hospital of Soochow University, Medical Center of Soochow University, Suzhou DushuLake Hospital, Suzhou, Jiangsu 215123 China; 2 Department of Vascular Surgery, Xiyuan Hospital of China Academy of Chinese Medicine Sciences, Suzhou Hospital, Suzhou, Jiangsu 215100 China; 3 Department of General Surgery, The Fourth Affiliated Hospital of Soochow University, Medical Center of Soochow University, Suzhou DushuLake Hospital, Suzhou, Jiangsu 215123 China; 4 Department of Urology, The Fourth Affiliated Hospital of Soochow University, Medical Center of Soochow University, Suzhou DushuLake Hospital, Suzhou, Jiangsu 215123 China

**Keywords:** Urinary bladder neoplasms, Embolization, Transurethral resection of bladder tumor, Large bladder tumor, Propensity score matching, Therapeutic

## Abstract

**Background:**

Transurethral resection of bladder tumor (TURBT) can be technically challenging in patients with large bladder tumors because intraoperative bleeding may obscure the surgical field, prolong the procedure, and hinder complete tumor resection. Preoperative bladder artery embolization may reduce tumor vascularity and facilitate subsequent TURBT; however, comparative evidence regarding its perioperative benefits and safety remains limited. This study evaluated the perioperative outcomes of preoperative bladder artery embolization followed by TURBT within 24 h in patients with bladder tumors measuring ≥ 3 cm.

**Results:**

Among 75 eligible patients, 27 underwent preoperative bladder artery embolization followed by TURBT and 48 underwent TURBT alone. Propensity score matching generated 17 matched pairs. Compared with the TURBT-alone group, the embolization group had significantly lower intraoperative blood loss (68.03 [56.68, 75.50] mL vs. 101.96 [93.62, 116.67] mL, *P* = 0.005) and shorter operation time (60.00 [49.00, 67.00] min vs. 80.00 [75.00, 87.00] min, *P* = 0.009). Postoperative hospital stay (7 [7, 8] days vs. 8 [8, 10] days, *P* = 0.035) and catheterization time (6 [6, 7] days vs. 8 [7, 8] days, *P* = 0.009) were also shorter in the embolization plus sequential TURBT group.

**Conclusions:**

For bladder tumors measuring ≥ 3 cm, bladder artery embolization followed by sequential TURBT within 24 h may reduce intraoperative blood loss, shorten operation time, and promote early postoperative recovery.

## Background

Bladder cancer is the most common malignant tumor of the urinary system and one of the major malignancies that seriously threaten public health. Transurethral resection of bladder tumors (TURBT), transurethral laser resection, and second resection procedures are common surgical approaches for early-stage bladder cancer. Among these, TURBT is considered the gold standard for diagnosing and treating early-stage bladder cancer and is widely employed in clinical practice [1, 2].

TURBT can be technically challenging in relatively large bladder tumors because bleeding may impair visualization. A diameter of ≥ 3 cm has been used to define large tumors in NMIBC risk stratification and previous studies of preoperative embolization [2, 3]. Therefore, in the present study, bladder tumors measuring ≥ 3 cm were defined as relatively large tumors. Furthermore, prolonged operative time—a consequence of compromised visualization—is associated with an elevated incidence of perioperative complications, including urinary tract infection, sepsis, pulmonary embolism, myocardial infarction, and increased mortality [2, 4, 5]. Selective arterial embolization (SAE) can reduce tumor vascularity and perioperative bleeding by selectively occluding tumor-feeding arteries [6]. In urologic practice, preoperative arterial embolization has been used to manage large hypervascular renal masses and to reduce prostate volume before surgery for benign prostatic hyperplasia [5, 7]. More recently, several studies have evaluated preoperative arterial embolization before TURBT. However, previous studies varied in patient selection and study design, including cohorts selected according to tumor stage or VI-RADS score, comparative cohorts with significant baseline differences in tumor size, and small uncontrolled series of very large NMIBC [2, 5, 8]. In addition, the interval between embolization and TURBT was not consistently standardized, and perioperative outcomes and embolization-related adverse events were variably reported.

In addition, attention should be paid to adverse events associated with embolization. Interventional procedures may result in access-site bleeding and post-embolization syndrome (PES), which is characterized by symptoms such as fever, nausea, abdominal pain, and fatigue. PES has been reported to typically occur within 24–72 h after embolization and may interfere with subsequent surgical treatment [9]. In our clinical experience, clinically relevant symptoms such as fever and nausea were commonly observed more than 24 h after embolization. Accordingly, we focused on patients who underwent TURBT within 24 h after completion of bladder artery embolization. Therefore, the present study used propensity score matching to compare perioperative outcomes and embolization-related adverse events between patients with bladder tumors ≥ 3 cm who underwent preoperative bladder artery embolization followed by TURBT within 24 h and those who underwent TURBT alone.

## Materials and methods

### Clinical data

**Fig. 1:**
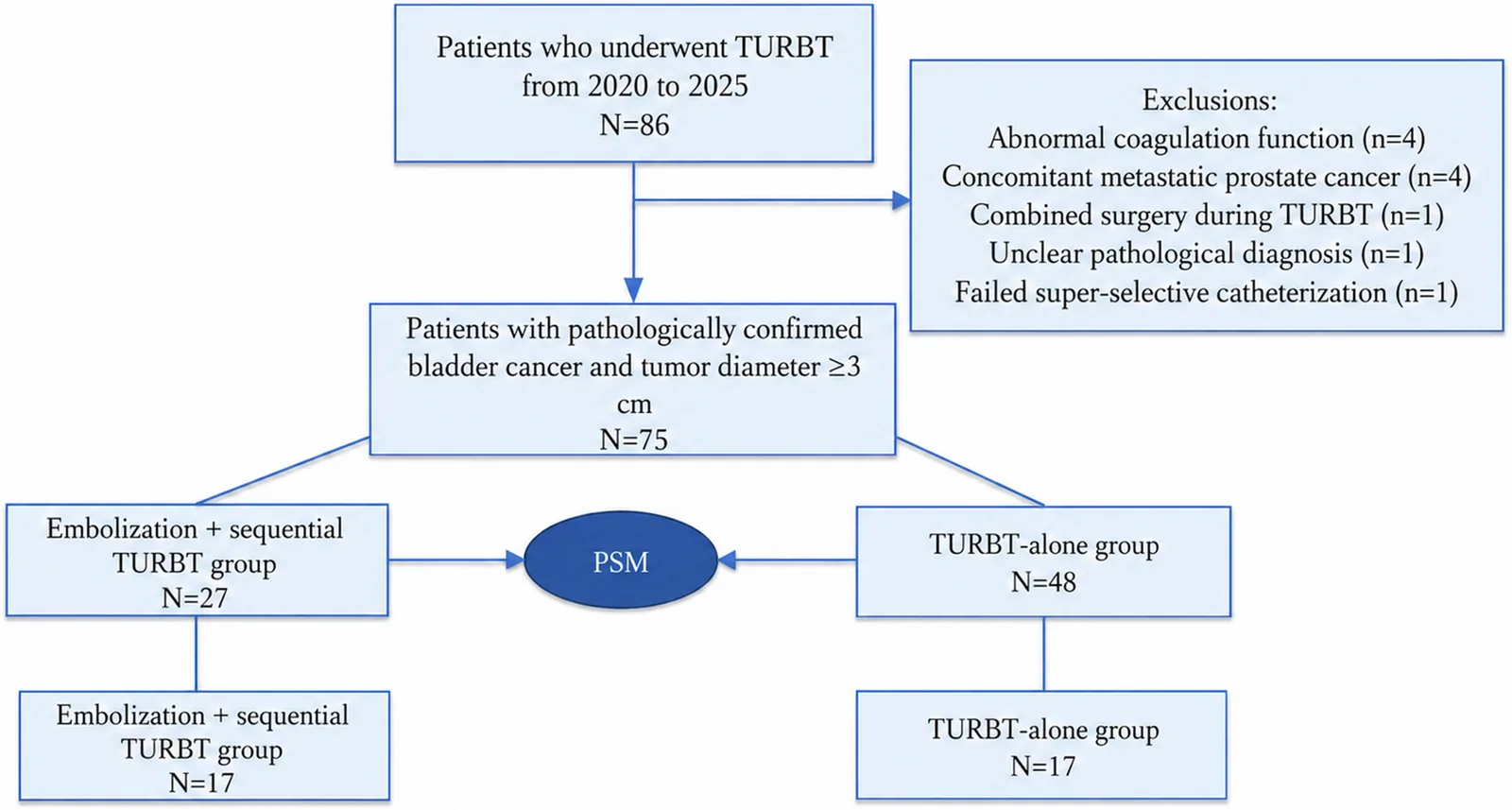
Flowchart of patient selection and propensity score matching

This single-center retrospective study included patients with pathologically confirmed bladder tumors measuring ≥ 3 cm who underwent TURBT between 2020 and 2025, during which preoperative bladder artery embolization was in use and the procedural protocol remained consistent. A total of 86 patients were initially screened. Eleven patients were excluded, including 4 patients with abnormal coagulation function, 4 patients with concomitant prostate cancer or metastatic disease, 1 patient who underwent another surgical procedure during TURBT, 1 patient with an unclear pathological diagnosis, and 1 patient in whom super-selective catheterization failed. The patient selection process is shown in Fig. 1. Patients were divided into two groups according to treatment strategy: those who underwent selective bladder artery embolization followed by TURBT within 24 h and those who underwent TURBT without preoperative embolization. The decision to perform preoperative bladder artery embolization was based on joint clinical assessment by the urologist and interventional radiologist, primarily when substantial bleeding or difficulty in achieving hemostasis during TURBT was anticipated. The inclusion criteria were as follows: (1) age ≥ 18 years; (2) pathologically confirmed bladder cancer; (3) maximum tumor diameter ≥ 3 cm; (4) treatment with TURBT; and (5) complete clinical and perioperative data. All included TURBT procedures were performed by urologists with more than 5 years of experience in endoscopic bladder tumor surgery. All bladder artery embolization procedures were performed by the same interventional radiologist, who had more than 10 years of experience in endovascular interventions. The exclusion criteria were as follows: (1) abnormal coagulation function; (2) concomitant other malignancies or metastatic disease; (3) current use of anticoagulant or antiplatelet drugs; (4) combined surgery during TURBT; (5) unclear pathological diagnosis; (6) failed super-selective catheterization or unsuccessful embolization; and (7) incomplete data.

### Equipment

Diagnostic angiography was performed using a 5-F Headhunter or Simmons angiographic catheter (APT Medical Inc., Shenzhen, China), and super-selective catheterization was performed using a Maestro microcatheter (Merit Medical Systems, Inc., South Jordan, UT, USA). The embolic agents were Merit Embosphere microspheres with diameters of 100–300 μm, 300–500 μm, and 500–700 μm, and Alicon gelatin sponge particles with diameters of 350–560 μm and 560–710 μm. Vascular closure devices included the Terumo Angio-seal VIP and Abbott Starclose SE.

### Surgical methods

TURBT-alone group: After anesthesia, patients were placed in the lithotomy position. Under direct vision, an F26 Stryker resectoscope was inserted into the bladder. TURBT was performed with a vaporization power setting of 90 W and a coagulation power setting of 100 W. The bladder tumor, together with approximately a 1-cm margin of surrounding mucosa, was resected down to the muscularis layer as completely as possible. After tumor removal and adequate hemostasis, an F18 three-way catheter was placed for continuous bladder drainage.

**Fig. 2:**
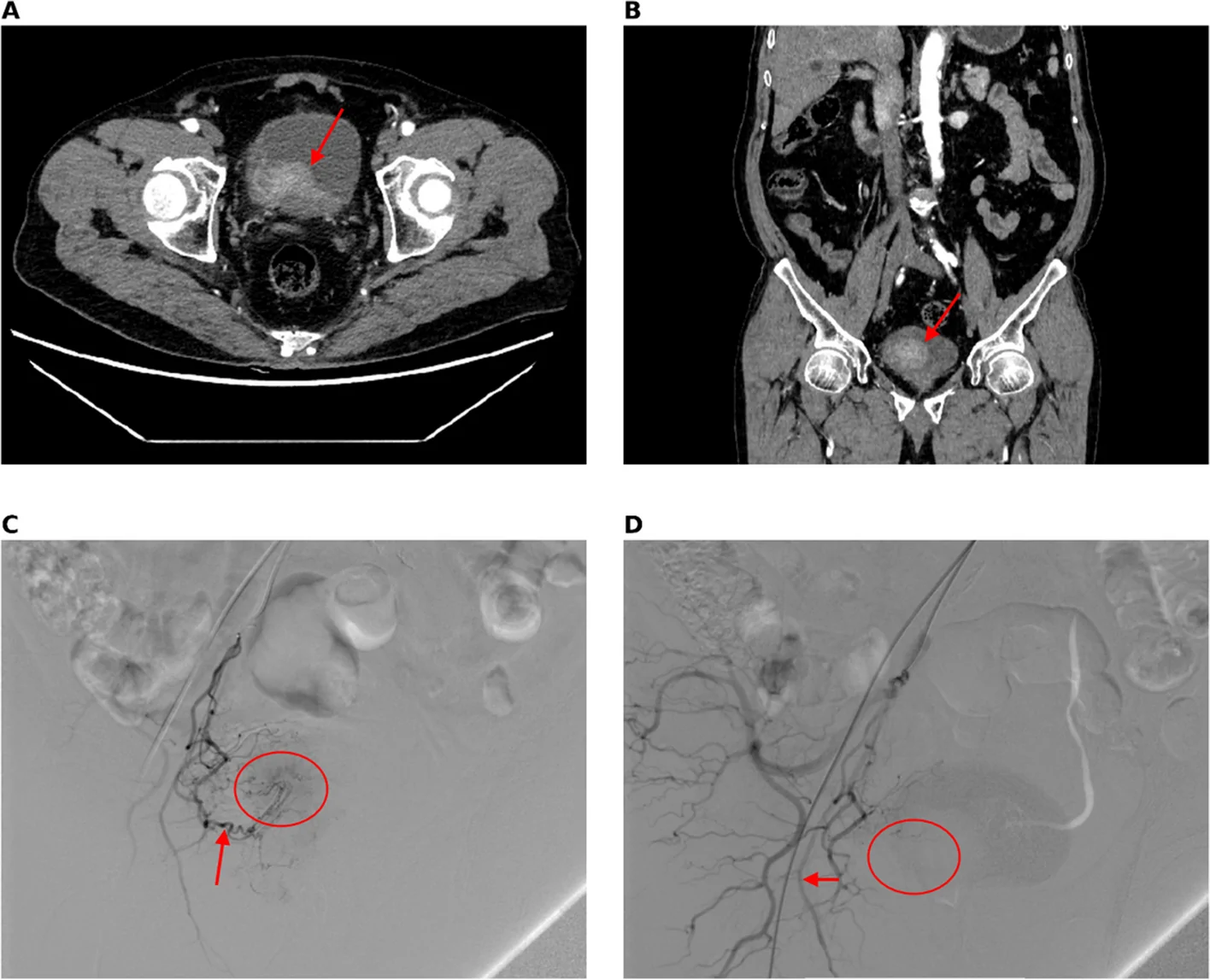
Representative imaging findings before and after bladder artery embolization. **A** Axial and **B** coronal contrast-enhanced CT images show a 5-cm bladder tumor with marked enhancement (red arrows). Postoperative histopathology revealed high-grade invasive urothelial carcinoma with lamina propria invasion, corresponding to at least pT1 disease. **C** Pre-embolization selective angiography demonstrates a tumor-feeding vesical artery (red arrow) and prominent tumor staining (red circle). A microcatheter was advanced super-selectively into the tumor-feeding vesical artery for embolization. **D** Completion angiography after embolization with 100–300 µm Embosphere microspheres and 350–560 µm gelatin sponge particles demonstrates near-complete disappearance of tumor staining in the corresponding region (red circle), while background pelvic arterial branches remain visible (red arrow)

Embolization plus sequential TURBT group: In this group, selective bladder artery embolization was performed before TURBT. Preoperative CT was reviewed to assess the bladder tumor and its extent, and a representative image is shown in Fig. 2A. Under local anesthesia, femoral artery puncture was performed, followed by bilateral internal iliac artery angiography to identify the feeding arteries of the bladder tumor (Fig. 2B). A microcatheter was advanced into the target vessels for super-selective embolization of the superior and inferior vesical arteries. In most patients, 100–300 µm particles were used. The interventional radiologist determined whether other particle sizes were needed according to the caliber of the tumor-feeding vessels and residual tumor staining; in some patients, larger particles were subsequently administered. The angiographic endpoint was defined as near-complete disappearance of tumor staining on final angiography (Fig. 2C). After embolization, the puncture site was closed using a vascular closure device. TURBT was performed within 24 h after embolization using the same technique as in the TURBT-alone group.

### Data collection

The following baseline variables were collected: age, sex, hypertension, diabetes mellitus, muscle-invasive status, prothrombin time, activated partial thromboplastin time, platelet count, body weight, serum creatinine, and tumor size. Tumor size was determined based on preoperative imaging or cystoscopic evaluation, and the maximum diameter was recorded. For patients with multiple lesions, the largest lesion was used to represent tumor size. The perioperative outcomes included intraoperative blood loss, operation time, postoperative hospital stay, and postoperative catheterization time. Intraoperative blood loss was estimated using the following formula: estimated intraoperative blood loss (mL) = (preoperative HCT − postoperative HCT)/preoperative HCT × body weight (kg) × 7% × 1000. Preoperative HCT and hemoglobin were defined as the most recent values measured before TURBT, whereas postoperative HCT and hemoglobin were defined as the first routine values measured within 24 h after TURBT, usually on postoperative day 1 [10, 11]. Embolization-related complications were graded according to the modified CIRSE classification system [12].

### Statistical analysis

Statistical analyses were performed using R software (version 4.4.1; R Foundation for Statistical Computing, Vienna, Austria). Propensity score matching was performed to reduce baseline imbalance between the embolization plus sequential TURBT group and the TURBT-alone group. Propensity scores were estimated using multivariable logistic regression with preoperative bladder artery embolization as the dependent variable, and the covariates included age, sex, hypertension, diabetes mellitus, muscle-invasive status, prothrombin time, activated partial thromboplastin time, platelet count, body weight, serum creatinine, and tumor size. Patients were matched at a 1:1 ratio using nearest-neighbor matching without replacement, with a caliper width of 0.2 standard deviations of the logit of the propensity score. Covariate balance was assessed using standardized mean differences, with an absolute standardized mean difference < 0.2 considered acceptable. After matching, perioperative outcomes were compared using the Wilcoxon signed-rank test because the paired differences were not normally distributed. A two-sided *P* value < 0.05 was considered statistically significant.

## Results

### Patient selection and baseline characteristics

**Fig. 3:**
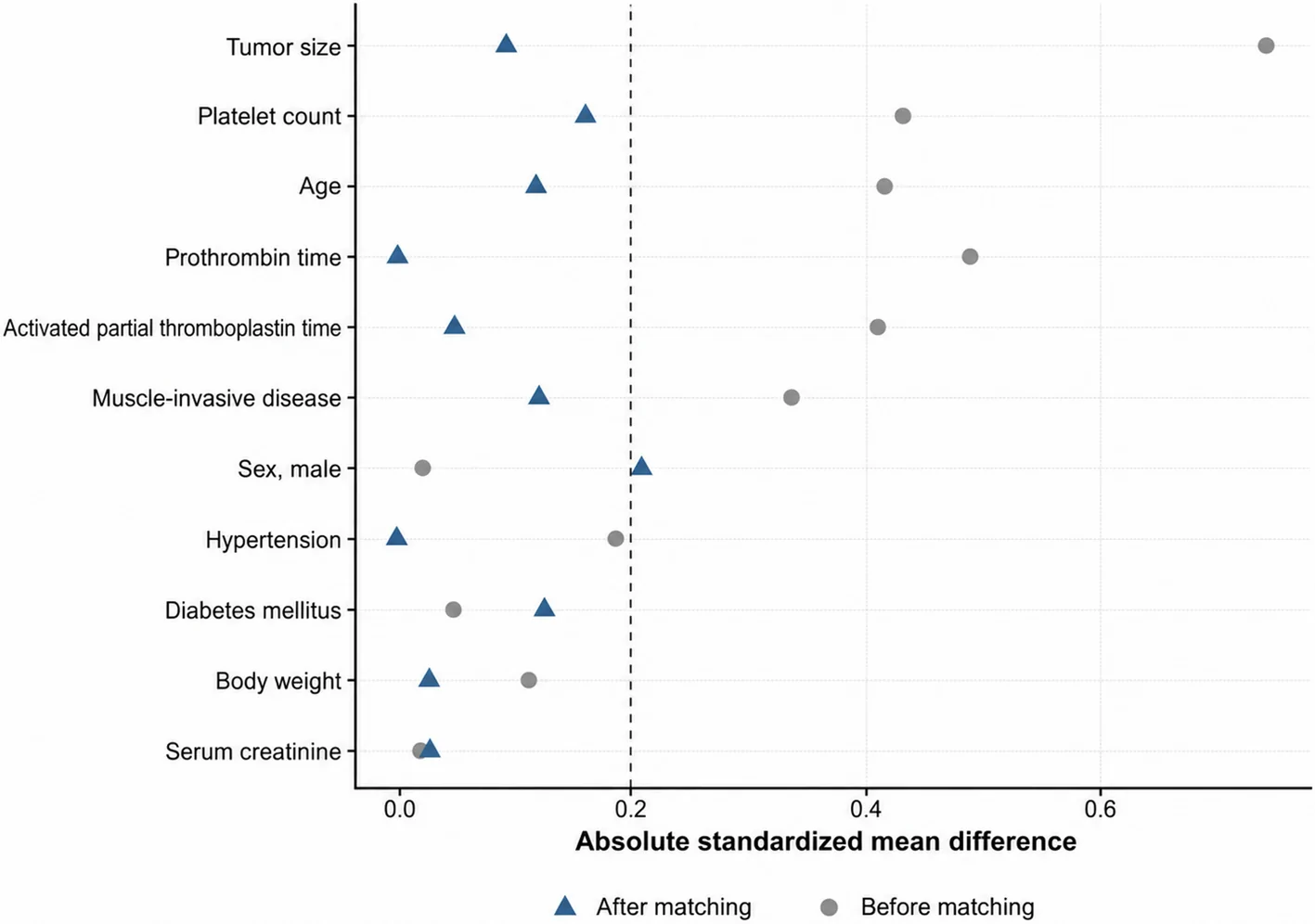
Covariate balance before and after propensity score matching. Absolute standardized mean differences before and after matching are shown. The dashed line indicates the balance threshold of 0.2

Finally, 75 patients were included in the analysis, including 27 patients in the embolization plus sequential TURBT group and 48 patients in the TURBT-alone group. After 1:1 propensity score matching, 17 matched pairs were generated. Baseline characteristics before and after matching are summarized in Table 1. Before matching, tumor size differed significantly between the two groups, whereas other baseline variables were generally comparable. After matching, baseline imbalance was reduced, as shown by the standardized mean differences listed in Table 2 and visually presented in Fig. 3. Most covariates showed improved balance after matching, although sex showed a marginal residual imbalance (absolute standardized mean difference = 0.209).

**Table 1:** Baseline characteristics before and after propensity score matching

	Before matching			After matching		
Characteristics	Embolization + TURBT (*n* = 27)	TURBT alone (*n* = 48)	*P*	Embolization + TURBT (*n* = 17)	TURBT alone (*n* = 17)	*P*
Age, median [IQR]	74.00 [66.00, 82.00]	68.50 [62.75, 75.25]	0.117	72.00 [67.00, 79.00]	74.00 [68.00, 81.00]	0.788
Sex, *n* (%)			1.000			1.000
Female	3 (11.1)	5 (10.4)		1 (5.9)	2 (11.8)	
Male	24 (88.9)	43 (89.6)		16 (94.1)	15 (88.2)	
Hypertension, *n* (%)			0.440			1.000
No	11 (40.7)	24 (50.0)		6 (35.3)	6 (35.3)	
Yes	16 (59.3)	24 (50.0)		11 (64.7)	11 (64.7)	
Diabetes mellitus, *n* (%)			1.000			1.000
No	22 (81.5)	40 (83.3)		12 (70.6)	11 (64.7)	
Yes	5 (18.5)	8 (16.7)		5 (29.4)	6 (35.3)	
Muscle-invasive disease, *n* (%)			0.165			1.000
No	13 (48.1)	31 (64.6)		7 (41.2)	6 (35.3)	
Yes	14 (51.9)	17 (35.4)		10 (58.8)	11 (64.7)	
Prothrombin time, median [IQR]	13.00 [12.65, 13.25]	13.25 [12.88, 13.80]	0.058	13.00 [12.70, 13.20]	13.00 [12.70, 13.30]	0.660
Activated partial thromboplastin time, median [IQR]	35.50 [34.45, 37.40]	34.70 [32.42, 36.52]	0.200	35.40 [33.00, 36.20]	35.40 [34.20, 36.50]	0.897
Platelet count, mean ± SD	229.74 ± 60.00	206.33 ± 48.01	0.089	218.29 ± 52.83	210.06 ± 49.83	0.599
Body weight, mean ± SD	65.94 ± 8.80	66.96 ± 9.34	0.638	66.49 ± 9.59	66.74 ± 7.75	0.909
Serum creatinine, mean ± SD	77.90 ± 10.61	78.12 ± 11.38	0.934	79.16 ± 10.67	79.47 ± 11.09	0.943
Tumor size, median [IQR]	5.00 [4.25, 6.00]	4.00 [3.50, 5.00]	0.003	5.00 [4.00, 5.00]	4.50 [4.00, 5.00]	0.699

Before matching, age, prothrombin time, activated partial thromboplastin time, and tumor size were compared using the Mann–Whitney *U* test; platelet count, body weight, and serum creatinine were compared using Welch’s two-sample *t*-test. Sex and diabetes mellitus were compared using Fisher’s exact test, whereas hypertension and muscle-invasive disease were compared using Pearson’s chi-square test. After matching, prothrombin time was compared using the Wilcoxon signed-rank test, and the remaining continuous variables were compared using the paired *t*-test. Binary categorical variables after matching were compared using exact McNemar’s test. Covariate balance was assessed using standardized mean differences

**Table 2:** Standardized mean differences of covariates before and after PSM

Variable	SMD_Before	SMD_After
Age	0.415	− 0.118
Sex	− 0.022	0.209
Hypertension	0.187	0
Diabetes mellitus	0.049	− 0.126
Muscle-invasive disease	0.336	− 0.121
Prothrombin time	− 0.488	0
Activated partial thromboplastin time	0.41	− 0.05
Platelet count	0.431	0.16
Body weight	− 0.113	− 0.028
Serum creatinine	− 0.02	− 0.029
Tumor size	0.738	− 0.092

An absolute SMD < 0.2 was considered acceptable balance. Sex showed a marginal residual imbalance after matching

*Abbreviations: PSM* propensity score matching, *SMD* standardized mean difference

### Perioperative outcomes after matching

**Fig. 4:**
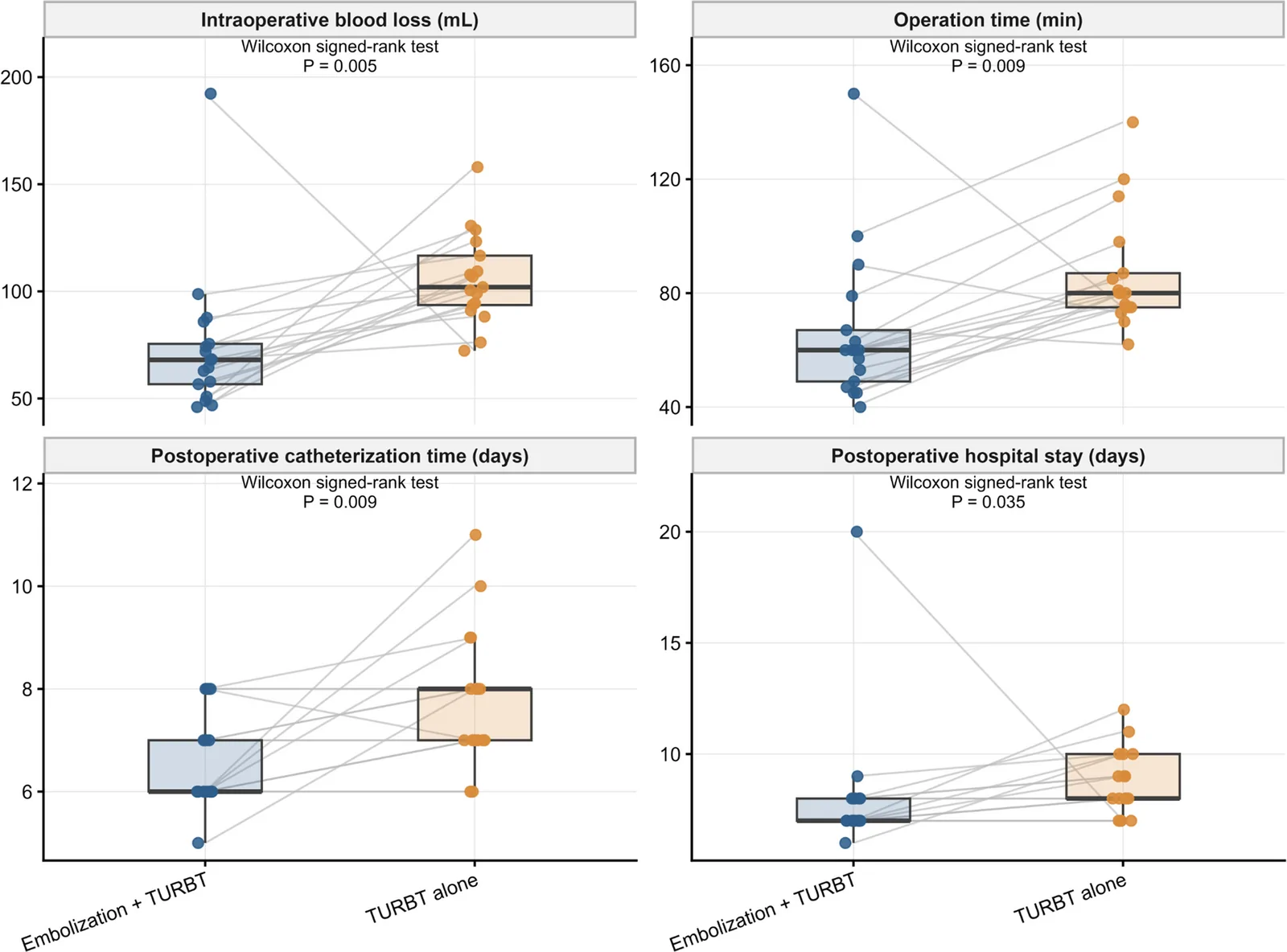
Matched-pair comparison of perioperative outcomes after propensity score matching. Gray lines connect matched pairs between the embolization plus sequential TURBT group and the TURBT-alone group. *P* values were calculated using the Wilcoxon signed-rank test

Perioperative outcomes after matching are presented in Fig. 4. Compared with the TURBT-alone group, the embolization plus sequential TURBT group had significantly lower estimated intraoperative blood loss (68.03 [56.68, 75.50] mL vs. 101.96 [93.62, 116.67] mL, *P* = 0.005), a smaller perioperative decrease in hemoglobin (0.66 [0.55, 0.79] g/dL vs. 1.08 [0.95, 1.26] g/dL, *P* = 0.003), shorter operation time (60.00 [49.00, 67.00] min vs. 80.00 [75.00, 87.00] min, *P* = 0.009), shorter postoperative hospital stay (7 [7, 8] days vs. 8 [8, 10] days, *P* = 0.035), and shorter postoperative urinary catheterization duration (6 [6, 7] days vs. 8 [7, 8] days, *P* = 0.009). No patient in the embolization plus sequential TURBT group required blood transfusion, whereas one patient (5.9%) in the TURBT-alone group required transfusion.

### Technical success and embolization-related adverse events

Among 28 patients in whom preoperative embolization was attempted, successful super-selective bladder artery embolization was achieved in 27 patients, yielding a technical success rate of 96.4%. Super-selective catheterization failed in one patient, and embolization could not be completed; this patient was therefore excluded from the comparative outcome analysis.

**Fig. 5:**
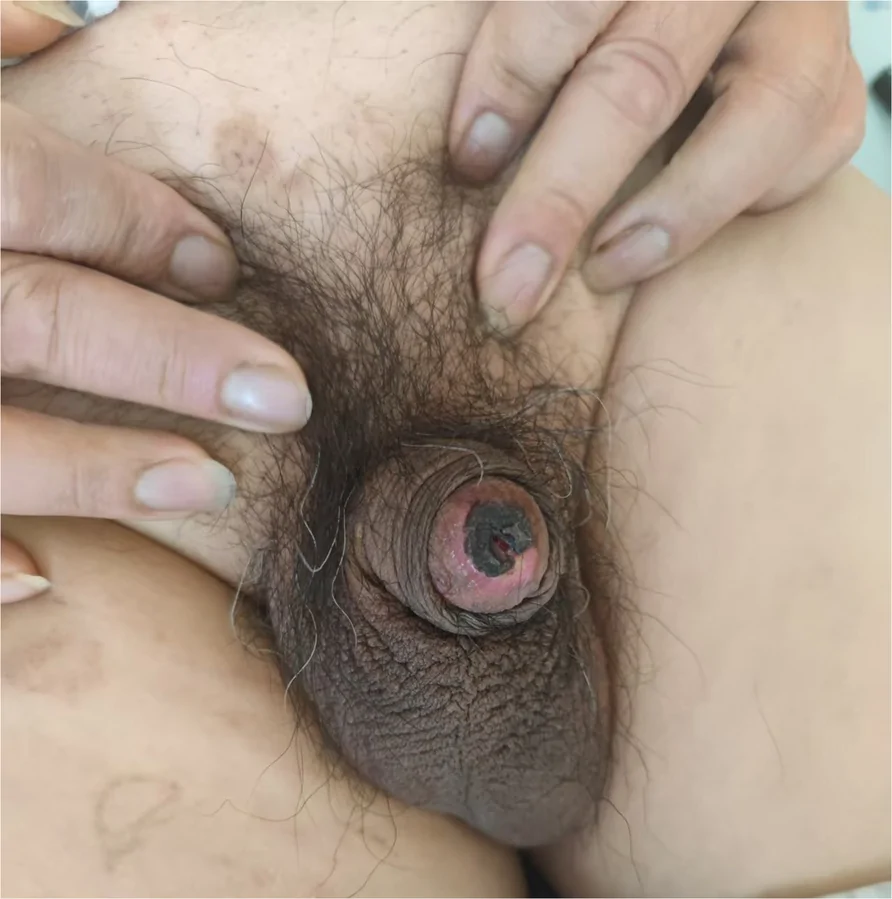
Embolization-related glans ischemic necrosis. After embolization with 100–300 µm and 300–500 µm Embosphere microspheres and 350–560 µm gelatin sponge particles, the patient experienced pain and ischemic necrosis of the glans penis, which improved after symptomatic treatment and restoration of blood supply. This complication resolved after conservative management, and no additional intervention was required

Among the 27 patients who underwent successful embolization, six patients experienced embolization-related complications, with overlapping events in some patients. Bladder spasm occurred in 4 patients (14.8%), fever in 3 (11.1%), and hematuria in 2 (7.4%). According to the modified CIRSE classification system, two patients had grade 2 complications and three had grade 3a complications. One additional patient experienced self-limited fever without additional treatment or delayed discharge. One of the grade 3a complications was glans skin ischemic necrosis, which improved after conservative treatment (Fig. 5).

## Discussion

TURBT remains central to the diagnosis, staging, and initial management of bladder cancer, particularly NMIBC [13]. Tumor size and number are established predictors of recurrence and progression [14–16], and larger tumors may also make endoscopic resection more challenging when intraoperative bleeding compromises visualization. Therefore, adjunctive strategies that reduce bleeding and facilitate tumor resection remain clinically relevant for patients with relatively large bladder tumors.

Super-selective vesical artery embolization has been used to control refractory hematuria in patients with bladder cancer [17]. In bladder cancer, pelvic arterial embolization has traditionally been applied mainly for the control of refractory hematuria. Previous studies have shown that embolization of the superior and inferior vesical arteries or internal iliac arterial branches can achieve a short-term hemostatic rate of 75%–100% [18]. However, the present study differs from emergency hemostatic embolization because embolization was performed preoperatively to reduce tumor blood flow before TURBT. Previous studies have reported favorable perioperative outcomes after preoperative arterial embolization before TURBT, including shorter operative time and smaller perioperative hemoglobin decreases [2, 8]. Bang et al. also demonstrated the feasibility of this approach in patients with large NMIBC tumors [5]. These findings suggest that selective bladder artery embolization may improve the safety and efficiency of TURBT in patients with relatively large bladder tumors. Compared with previous studies, the present study included patients with bladder tumors measuring ≥ 3 cm and used PSM to reduce baseline imbalance, thereby providing additional comparative evidence supporting the perioperative value of preoperative bladder artery embolization. Most covariates showed improved balance after matching, and the prematching imbalance in tumor size was substantially reduced, addressing a limitation of previous retrospective comparisons. As shown in Table 2 and Fig. 3, although the SMD for sex remained 0.209, the overall comparability of the matched cohort was considered acceptable [19, 20].

**Fig. 6:**
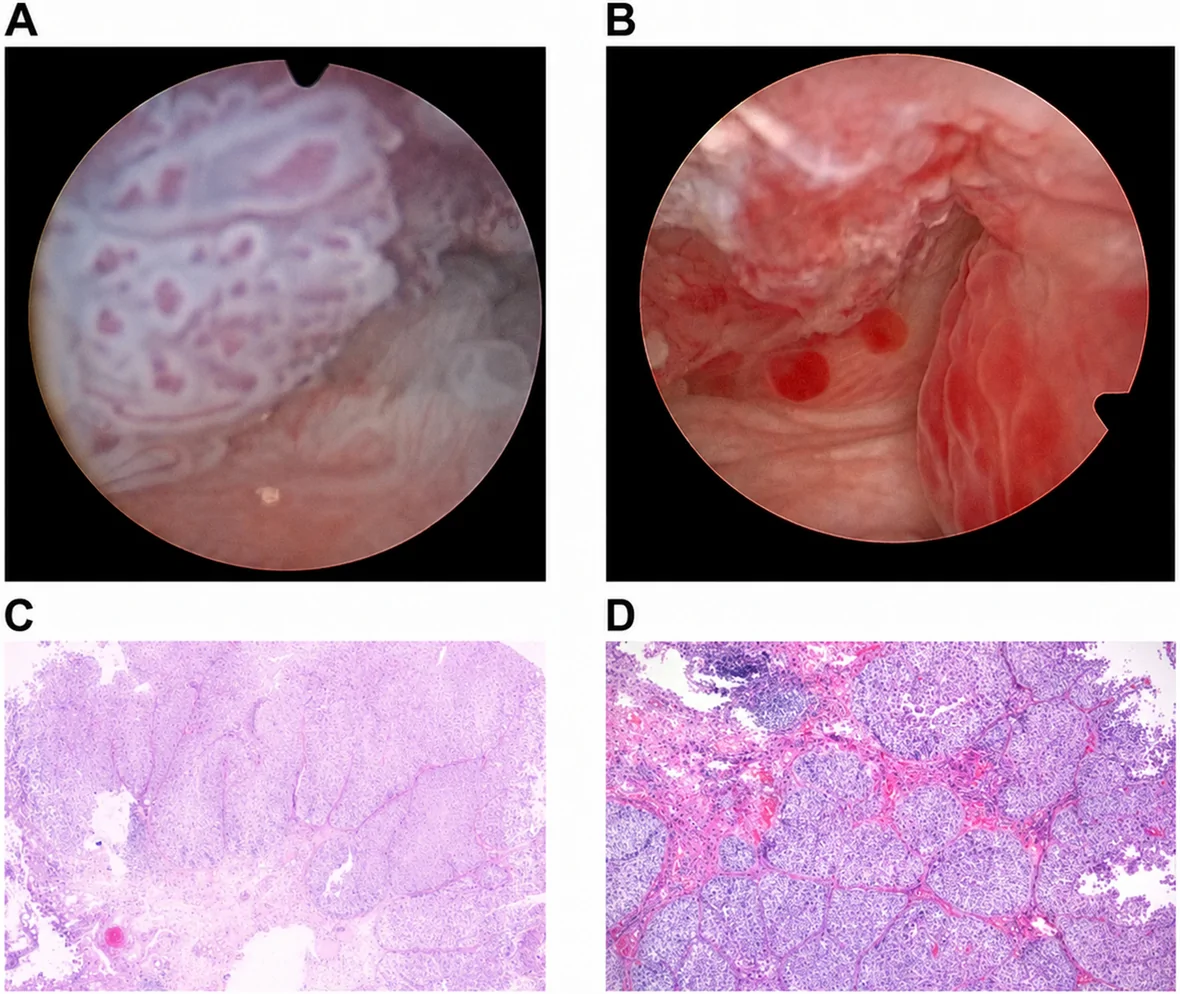
Representative cystoscopic and histopathologic findings with and without preoperative bladder artery embolization. **A** In a patient who underwent preoperative bladder artery embolization, the tumor surface appeared pale with reduced visible vascularity during TURBT. **B** In a patient who did not undergo preoperative embolization, the tumor showed marked hyperemia and abundant superficial vessels during TURBT. **C** Representative histopathologic image from an embolized tumor shows less conspicuous intratumoral vascularity and vascular congestion. **D** Representative histopathologic image from a nonembolized tumor shows more prominent intratumoral vascular channels and vascular congestion

The rationale for preoperative embolization was supported by angiographic, cystoscopic, and histopathologic findings. In this study, preoperative CT demonstrated the tumor burden, angiography before embolization showed tumor-feeding arteries and tumor staining, and post-embolization angiography confirmed near-complete disappearance of tumor staining (Fig. 2). By reducing tumor arterial inflow before endoscopic resection, bladder artery embolization may provide a clearer operative field. By reducing tumor arterial inflow before endoscopic resection, bladder artery embolization may reduce tumor vascularity and facilitate intraoperative hemostasis. In addition to the angiographic findings, cystoscopic and histopathologic observations provided complementary support for the hemostatic effect of embolization. In patients who underwent preoperative embolization, ischemic changes and reduced vascularity of the tumor were observed, whereas tumors in patients without embolization showed more abundant blood supply (Fig. 6). The tumor boundary appeared more clearly distinguishable from the surrounding bladder mucosa after embolization; however, this observation was based on qualitative assessment without a standardized scoring system. These visual findings are consistent with the observed reduction in intraoperative blood loss and operation time in the embolization plus sequential TURBT group. During the procedure, the use of small-diameter embolic particles and microcatheter-based super-selective catheterization may help reduce the risk of embolic particle reflux. Particle-size selection requires a balance between adequate embolization of the tumor-feeding arteries and minimizing the risks of proximal occlusion, incomplete tumor devascularization, bladder wall ischemia or infarction, and reflux-mediated non-target embolization. In our practice, embolization was performed slowly under real-time fluoroscopic guidance, with the embolic material adjusted dynamically according to the caliber of the tumor-feeding vessels and residual tumor staining on angiography.

The interval between embolization and TURBT is also an important consideration. Common complications of bladder artery embolization include postembolization syndrome, bladder necrosis, skin necrosis, pelvic weakness, and puncture-site bleeding [21]. Severe postembolization syndrome or puncture-site complications may delay subsequent treatment and increase perioperative risk. Therefore, performing TURBT within 24 h after embolization may help preserve the hemostatic effect while reducing the likelihood that clinically apparent postembolization symptoms interfere with the surgical workflow. In the present study, most embolization-related complications were manageable, with two patients classified as modified CIRSE grade 2 and three as grade 3a; no grade 3b–6 complications occurred. In addition, vascular closure devices (VCDs) were used for femoral puncture-site management. Existing evidence suggests that, compared with manual compression, VCDs can improve patient comfort and satisfaction and shorten hemostasis time and lower-limb immobilization time [22]. All patients who received sequential treatment underwent TURBT within 24 h after embolization, and no puncture-site bleeding was observed. In the present study, one patient developed ischemia and necrosis after embolization, which may have been related to the use of an embolic material with an excessively large diameter that failed to enter the target vessel, resulting in partial reflux of embolic particles into adjacent arterial branches. This highlights the need for further investigation into the effects of embolic material diameter and embolization technique on procedural outcomes. Intraoperative cone-beam CT (CBCT) may also be used to identify potential vascular anastomoses and adjacent organ blood supply, thereby helping to avoid nontarget embolization and optimize catheter placement [23].

Estimated intraoperative blood loss was statistically lower in the embolization group, although the absolute between-group difference was modest. We considered that the HCT-based formula provides only an indirect estimate of blood loss and may not fully reflect the actual magnitude of intraoperative bleeding. We therefore also compared the perioperative decrease in hemoglobin between the two groups, which provided additional evidence of reduced perioperative blood loss. Although an approximately 20-min reduction in operation time and a modest reduction in estimated intraoperative blood loss alone may not be sufficient to justify preoperative embolization, its potential clinical value should be considered together with the smaller perioperative decrease in hemoglobin, potentially reduced procedural risk, shorter postoperative hospital stay, and shorter urinary catheterization duration observed in the embolization group. These combined benefits may be particularly relevant for patients with relatively large bladder tumors, especially those with poor general condition or an anticipated higher risk of perioperative bleeding, as they may reduce the burden of postoperative management and improve patient comfort. In addition, shorter postoperative hospitalization may reduce hospitalization costs and improve healthcare resource utilization.

This study has several limitations. First, this was a retrospective single-center study, and treatment allocation was based on clinical judgment rather than predefined criteria or randomization. Although propensity score matching was performed to reduce baseline differences, selection bias and residual or unmeasured confounding could not be completely eliminated. Second, the sample size was relatively small, especially after matching, which limited the statistical power and made perfect covariate balance difficult to achieve. Third, this study mainly focused on short-term perioperative outcomes, including intraoperative blood loss, operation time, postoperative hospital stay, and postoperative catheterization time. Long-term oncological outcomes, including recurrence, progression, re-TURBT findings, and bladder-preservation outcomes, were not fully assessed. Fourth, although all procedures were performed by experienced physicians from the same institution, operator-related variation and incomplete recording of some embolization-related procedural details could not be fully excluded because of the retrospective design. Further multicenter prospective studies are needed to validate the safety and efficacy of this sequential strategy and to evaluate its broader clinical value, including long-term oncological outcomes, cost-effectiveness, and patient-reported outcomes such as postoperative pain, recovery speed, and quality of life.

## Conclusions

Preoperative bladder artery embolization followed by sequential TURBT within 24 h was associated with reduced intraoperative blood loss, shorter operation time, and improved early postoperative recovery in patients with bladder tumors measuring ≥ 3 cm. This approach may be a feasible adjunctive strategy for facilitating TURBT in selected patients with relatively large bladder tumors, although further prospective validation is needed.

## Data Availability

The datasets used and/or analyzed during the current study are available from the corresponding author on reasonable request.

## Abbreviations

CBCT: Cone-beam computed tomography
CT: Computed tomography
HCT: Hematocrit
IQR: Interquartile range
MIBC: Muscle-invasive bladder cancer
NMIBC: Non-muscle-invasive bladder cancer
PES: Post-embolization syndrome
PSM: Propensity score matching
SAE: Selective arterial embolization
SD: Standard deviation
SMD: Standardized mean difference
TURBT: Transurethral resection of bladder tumor
VCD: Vascular closure device
